# Female-friendly residential facilities and living satisfaction: evidence from Yangpu District, Shanghai

**DOI:** 10.3389/fpubh.2025.1653419

**Published:** 2025-12-03

**Authors:** Zhiguo Fang, Jiachen Yao, Jianing Shi

**Affiliations:** College of Publishing, University of Shanghai for Science and Technology, Shanghai, China

**Keywords:** women-friendly, supporting facilities, residential satisfaction, subjective perception, Yangpu District

## Abstract

**Introduction:**

With the acceleration of global urbanization, the development of female-friendly cities has become a key objective in urban planning. This study investigates strategies for optimizing residential community facilities in Yangpu District, Shanghai, from a female-friendly perspective, aiming to inform policymakers and improve the quality of life and satisfaction of women.

**Methods:**

Drawing on field surveys conducted across 36 communities and 923 valid questionnaires, descriptive statistical analysis and structural equation modeling (SEM) were employed to examine women’s daily needs regarding safety, convenience, and opportunities for social interaction.

**Results:**

The key findings reveal that, first, the built environment of community facilities significantly influences perceptions of female-friendliness and residential satisfaction, with recreational facilities exerting the strongest impact. Second, the direct relationship between female-friendliness and satisfaction is relatively weak, potentially due to individual differences and variations in environmental contexts. Finally, subjective perception mediates the relationship between the built environment and overall satisfaction.

**Discussion:**

Based on these findings, this study proposes a comprehensive strategy: implementing incentive policies and evaluation mechanisms; optimizing spatial layouts, establishing women-specific facilities, and enhancing connectivity; and strengthening facility management and service standards.

## 1 Introduction

The pursuit of gender equality and inclusive urban development has propelled the concept of “women-friendly cities” to the forefront of global urban planning agendas. With the acceleration of global urbanization, creating urban environments that cater to the diverse needs and safety concerns of women has become a critical goal for sustainable and equitable development (1). This movement recognizes that traditional urban planning often overlooks the distinct experiences and requirements of women in public and residential spaces, potentially leading to inconvenience, reduced mobility, and diminished quality of life (2, 3).

Pioneering efforts, such as Seoul’s “Women-Friendly City Plan” launched in 2007 (4) and Vienna’s long-standing focus on infrastructure transformation considering women and children’s needs since the 1990s (5), exemplify this global trend. In China, Changsha emerged as the first city to explicitly propose building a “women-friendly city” in 2021, prioritizing women’s safety through tangible measures like enhanced street lighting and surveillance systems (6). Subsequently, major cities including Hong Kong, Shenzhen, and Shanghai have also expressed commitments to fostering women-friendly urban environments (7–9). This concept can to a certain extent alleviate gender differences in urban Spaces, aiming to integrate women’s daily life needs, including safety, convenience, social interaction, health and care, into the design and planning of residential areas, in order to create a safer, more comfortable and more inclusive living environment (6, 10, 11).

However, significant research gaps still exist, especially in the context of China, regarding the subtle mechanisms between the built environment of residential facilities and women’s experiences. Specifically, insufficient focus on facility-user relationship. While the importance of women-friendly facilities is acknowledged, empirical research specifically examining how the built environment of community facilities directly influences perceived women-friendliness and, ultimately, residential satisfaction remains relatively scarce, especially at the micro-scale and within the Chinese context (2). Furthermore, limited understanding of subjective perception’s role. The potential mediating role of residents’ subjective perception between the built environment and overall residential satisfaction, particularly from a gendered perspective, is underexplored. More importantly, heterogeneity of women’s needs: Existing studies often treat “women” as a monolithic group, lacking granular analysis of how diverse factors shape distinct facility preferences and satisfaction drivers within residential areas (10).

Therefore, this study aims to explore the role of the built environment of residential area supporting facilities, including transportation, residence, commerce, culture, leisure, etc., in influencing women’s perception of friendliness, and ultimately the causal path that affects women’s residential satisfaction. Identify and compare the relative attractiveness of different areas and community facilities within the community to women. Examine the specific relationship between female residents’ perception of female-friendliness and overall residential satisfaction. To explore the mediating role of subjective perception between facility environment and residential satisfaction. Taking Yangpu District of Shanghai as an example, evidence-based strategies for strengthening female-friendliness in urban residential area planning and facility provision in China are proposed.

Yangpu District in Shanghai has become an ideal research case due to its unique historical heritage, economic vitality, large female population, diverse family structures, diverse urban structures (coexistence of old and new communities), and relatively developed transportation infrastructure. This diversity provides a solid research basis for studying the subtle needs of different women groups and the effectiveness of existing facility regulations (12).

This study theoretically deduces the understanding of the complex connection between the built environment of facilities and female-friendliness, subjective perception, and residential satisfaction through clear modeling and empirical testing. In terms of methods, a strict mixed approach is adopted, especially centering on the perspectives of women from different communities in major Chinese cities. New insights have emerged into the relative importance and appeal of different types of facilities to women. The research findings can provide evidence-based guidance for urban planners and decision-makers to optimize living facilities, enhance women’s safety and satisfaction, and build fair and women-friendly cities. Overall, this study provides an empirical basis for transforming the concept of female-friendly cities into practical improvements in women’s daily living.

## 2 Literature review

### 2.1 Residence satisfaction

With the acceleration of urbanization, the construction of community public facilities has become one of the important indicators to measure the quality of urban development. Existing literature indicates that community public facilities are an important factor influencing residential satisfaction, and there is a close theoretical connection between their construction level and the quality of residents’ lives (13–17). Wells and Yang (16) et al. found that well-designed public facilities can encourage residents to walk, increase physical activity, and enhance social interaction among residents during walking, thereby positively influencing residential satisfaction. A large amount of survey data further reveals that the completeness of infrastructure, the accessibility of public service facilities, and the diversity of recreational facilities are all significantly positively correlated with residential satisfaction (15, 17).

It is worth noting that residents’ demographic characteristics such as age, education level, and family income play a moderating role in the relationship between public facilities and residential satisfaction, and different characteristic groups have obvious differences in their demand preferences and satisfaction perceptions of public facilities (13, 14). However, most studies treat the community as a whole and do not fully consider the heterogeneity of different areas within the community. In addition, large-scale comparative studies across regions and multiple communities are relatively rare, which is not conducive to summarizing the general laws and special cases of the relationship between public facilities and residential satisfaction in different regional contexts. Therefore, in the process of continuous advancement in the construction of community public facilities, a deeper understanding of the complex relationship between them and residential satisfaction is of great academic value and practical guiding significance for optimizing community planning and improving the quality of residents’ lives.

### 2.2 Female friendliness

The concept of female-friendliness is grounded in environmental psychology and gender-sensitive urban planning theories, which emphasize that a female-friendly environment must address both objective safety and subjective comfort (3). In the context of urban community construction, the degree of female-friendliness has received extensive attention. Many scholars have pointed out that a female-friendly community is reflected in multiple aspects (18–21).

Firstly, the advancement of urban planning can help improve women’s safety. It is necessary to conduct human-scale design based on the diverse needs of women, provide more public spaces and community facilities, and increase the breadth and depth of women’s activity spaces, such as well-lit streets and mother-and-baby rooms, which can enhance the safety of the city and increase women’s sense of security (18, 20). Night lighting is operationalized as an indicator of female-friendliness because it directly influences women’s perception of safety during evening activities (18). However, current research on spatial planning lacks quantitative studies on how to accurately measure the specific needs of women in different regions for the breadth and depth of activity spaces, resulting in low utilization rates of some facilities in actual use.

What’s more, community services should cover projects such as women’s vocational training and mental health counseling, and enhance women’s sense of community belonging through various activities to support their self-development and social integration (21). However, existing women’s vocational training and mental health counseling projects are lacking in terms of service continuity and targeting, and the special needs of women of different ages and social strata have not been fully met.

In addition, the government encourages women to participate in community decision-making and guarantees their right to speak in community affairs, ensuring that community construction fully considers the female perspective (19). However, the design of specific participation mechanisms is not perfect, and there is a lack of effective supervision and feedback mechanisms to ensure the true implementation of women’s right to speak.

Therefore, building a female-friendly urban community is an important foundation for achieving sustainable urban development and social harmony.

*H1*: The friendliness of women in the community has a significant positive impact on residential satisfaction.

### 2.3 Facility built environment

Community facility construction is a key element in enhancing the quality of life for residents and promoting community development, attracting in-depth research from scholars in various fields (22–26). The suitability of public facilities not only depends on residents’ needs and the convenience of use but also on the operational efficiency and maintenance costs of the facilities themselves. Latham and Layton (24) and others pointed out that social infrastructure in cities (libraries, parks, commercial places, transportation facilities etc.) is crucial for public life, as it promotes social connections and fosters a sense of community belonging. In terms of analyzing the spatial supply capacity of public service facilities, community facilities are classified into three major categories: living services and commercial facilities, cultural and recreational facilities, and public transportation facilities, as the key research objects. Through specific spatial analysis methods, it was quantitatively evaluated that some community living services and commercial facilities are concentrated in the core area, while the periphery is lacking, resulting in significant differences in the convenience of residents accessing daily services (23). Cultural and recreational facilities do not match the age structure and cultural preferences of residents, failing to meet their diverse spiritual and cultural needs (25). The setting of public transportation facilities and the planning of routes do not well connect with and transfer in the hotspots of residents’ travel, affecting transportation efficiency and travel experience (26). However, in terms of research depth, there is a lack of in-depth analysis on how the built environment of facilities specifically affects the friendliness toward women, especially at the micro level, such as the impact of facility design details on women’s sense of security and comfort.

It is worth noting that the built environment of facilities plays a crucial role in creating a female-friendly space. However, existing research on how to better integrate women into social life from multiple dimensions such as safety assurance, environmental comfort, and inclusive atmosphere is relatively scarce. At the same time, high-quality infrastructure, rich public services, and a good ecological environment can enhance the convenience and comfort of residents’ lives, thereby improving their satisfaction with living; conversely, it can lead to residents’ dissatisfaction and lower their satisfaction with living (22).

It should be pointed out that the quality of the built environment of community facilities has a significant impact on residents’ subjective perception, the friendliness toward women, and their satisfaction with living to a certain extent. This assumption provides a theoretical basis and research guidance for further in-depth studies on the intrinsic connection between community facility construction and the improvement of residents’ quality of life.

*H2*: The built environment of the facility has a significant positive impact on the friendliness of women in the community.

*H3*: The built environment of the facility has a significant positive impact on subjective perception.

*H4*: The built environment of the facility has a significant positive impact on residential satisfaction.

### 2.4 Subjective perception

In the context of social public facility construction, research on the subjective perception of the public has shown a diverse trend of development (27–29). From the perspective of convenience perception, the satisfaction of public space is closely related to factors such as the convenience of public facilities, which is helpful for exploring the demands of community residents for the convenience of public facilities and the construction of an evaluation system (27). However, existing research lacks perception studies specifically targeting the female group.

Perception of accessibility involves physical distance, ease of access, and cost, which is particularly crucial for vulnerable groups (30, 31). Poor accessibility can lead to social equity issues. Wang et al. (28) found that the urban land accessibility of public service facilities is normally distributed, and the accessibility of different types of facilities varies significantly. Facility type has a significant impact on urban land accessibility, while different towns have no significant impact on the accessibility of educational facilities but have a significant impact on the accessibility of primary medical facilities. However, there is a lack of long-term tracking and evaluation of the effectiveness of accessibility improvement measures, making it difficult to determine whether these measures can continuously meet the needs of residents. Perception of aesthetics plays an important role in shaping the unique image of a city and improving the quality of life of residents. Wang et al. (29) evaluated the quality of the visual environment in communities by using time series street view images, including six perception indicators such as aesthetics and assigning scores. They found that improving the visual environment quality, including community aesthetics, enhances residents’ subjective perception and helps the government improve policies. However, existing research lacks unified standards for quantifying aesthetics, resulting in poor comparability of results among different studies.

These subjective perception dimensions are interrelated and jointly reflect the public’s attitude and experience toward public facilities, providing a reference basis for the planning, construction, and optimization of public facilities (32). Many studies have deeply analyzed the factors and their influences in each dimension from different cases and perspectives to enhance the social benefits and public satisfaction of public facility construction.

*H5*: Subjective perception has a significant positive impact on residential satisfaction.

**Figure 1:**
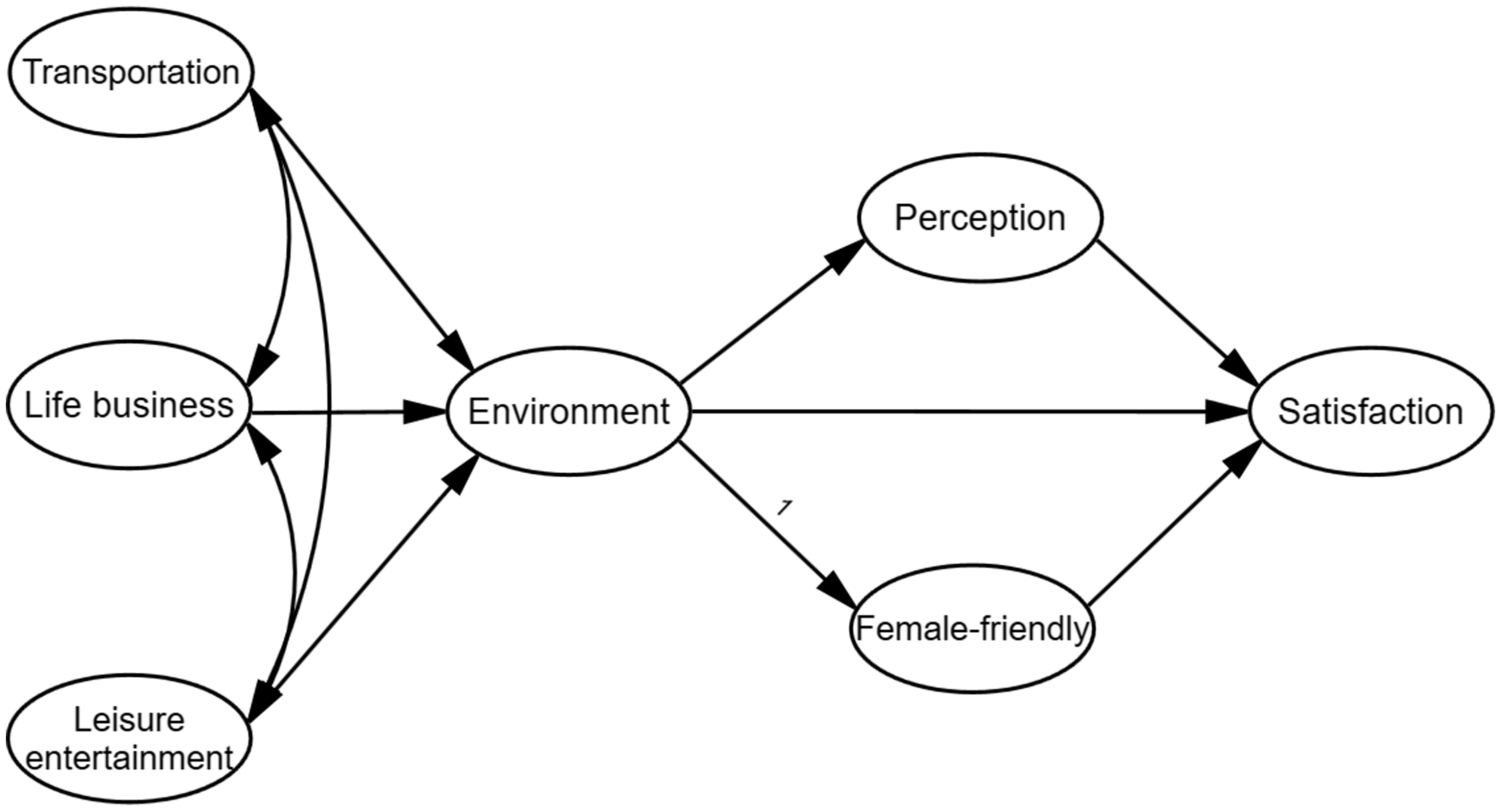
The structural model assumes the path.

Through the study of the literature, the following deficiencies were found. Firstly, is there a correlation mechanism between the built environment of facilities and the needs of women? Although most studies recognize the importance of female-friendly facilities, they lack micro-empirical analyses on how the built environment of facilities specifically affects female-friendly perception at the community scale and thereby influences residential satisfaction. Such studies are particularly scarce in the urban context of China. Secondly, whether there are mediating variables between the built environment of the facilities and satisfaction. Most existing studies focus on the direct correlation between the objective attributes of facilities and satisfaction, but rarely explore the transmission effect of residents, especially women’s subjective perception, in this regard. Thirdly, the heterogeneity of the needs of the female group has been simplified. Existing studies often view “women” as a single group and fail to adequately analyze the impact of differences such as age, family structure, and income on facility preferences and satisfaction drivers, resulting in limited explanatory power of research conclusions for the diverse needs of women. Therefore, in response to the above questions and deficiencies, this study constructed a theoretical model consisting of five hypotheses to systematically explore the causal relationships among the variables (Figure 1).

## 3 Research design and methods

### 3.1 Research site and data source

This study chose Yangpu District of Shanghai as the research site because its unique characteristics make it relevant and typical for research from a female-friendly perspective. Firstly, in terms of policy relevance. Yangpu District is one of the important areas in Shanghai that is committed to building a “women-friendly city.” Secondly, there is a larger female population and the social economy is diverse. This district has a large female population, including a considerable number of working professionals, students and older women, etc. Meanwhile, the diversity of age, occupation, family structure and income level is crucial for capturing the heterogeneity of women’s demands and experiences for residential facilities. Secondly, urban form and residential types. Yangpu showcases a unique integration of urban textures, including historical industrial heritage, traditional Shikumen and workers’ new villages, as well as modern high-rise residential developments (12). In addition, economic vitality and infrastructure. As a vibrant and revitalized area, Yangpu boasts significant commercial centers, educational institutions, cultural venues and residential areas. Relatively developed transportation infrastructure provides convenience for daily travel. This background reflects the complex interaction between residential facilities and the broader urban functions that shape women’s daily lives, safety and convenience.

**Figure 2:**
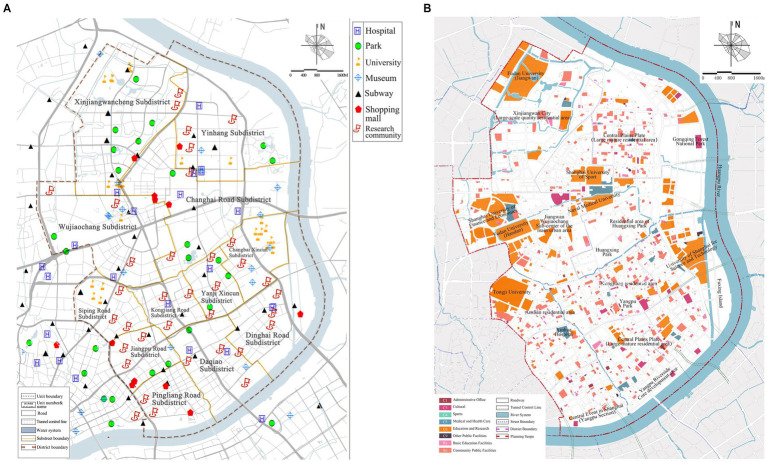
**(A)** Distribution of facilities and research sites in Yangpu District. **(B)** Public facilities planning land use in Yangpu District.

As shown in Figure 2A, due to the availability of data, this study mainly focuses on 36 residential communities in Yangpu District. The study area is about 198 hectares, the permanent population is about 152,500, and the population density is about 12.98 people/square meter. These communities are selected as research samples because their location in the street is significantly representative and their attribute characteristics are unique, which is convenient for in-depth exploration of the relationship between residents’ demand and the supply of public facilities space. As shown in Figure 2B, the schematic diagram of the planned land use for public facilities in Yangpu District is presented. From the perspective of spatial layout, the core community and the fringe community have different needs for transportation and other facilities. In terms of population structure, communities dominated by young groups and older children have obvious differences in demand for entertainment, fitness, senior support services, education and other facilities. The obvious difference between these communities and other areas in the street in all aspects has significant and special value for studying the matching degree and gap between residents’ demand and public facilities supply.

A total of 968 questionnaires were distributed in this survey, and 923 valid questionnaires were retrieved, with an effective rate of 95.4%. The questionnaires mainly covered multiple aspects such as environmental factors in residential areas, the current usage and satisfaction of public service facilities, and the demand for types of public service facilities. They involved different types of communities and people of various age groups within the research scope, with an average age of 33.95 years. This sample size is sufficient for SEM analysis because it exceeds the usually recommended ratio of 10 to 20 observations per estimated parameter (33). This ensures robust model estimation and reliable hypothesis testing.

### 3.2 Research methods

This study uses field investigation and questionnaire interview methods, combined with Autonavi map, POI data and structural equation model (SEM), to deeply explore the matching relationship between the current community residents’ demand and the space supply of public service facilities within the research scope, and serves as a reference for the accurate adjustment and optimization of public service facilities in the living circle of community.

IBM SPSS Amos 28.0 software was used to complete the path diagram of the structural equation model (SEM). In addition to direct effects, indirect effects were estimated using the Bootstrap method with 5,000 resamples to assess the mediating role of subjective perception (SP) between the built environment (BE) and residential satisfaction (RS). The significance of indirect effects was determined by 95% bias-corrected confidence intervals (CIs). Structural equation model consists of two parts: structural model and measurement model. The measurement model represents the correlation between explicit variables and corresponding latent variables, while the structural model represents the potential causality between endogenous variables and exogenous variables. As shown in Figure 2, the structural model is formed by the structural relationship composed of eight measurement models.

Structural equation models are generally represented by three matrix equations:


$$X=\Lambda_{x}\;\xi +\delta$$ (1)

$$Y=\Lambda_{y}\;\eta +\varepsilon$$ (2)

$$\eta =\beta_{\eta }+\Gamma \xi +\zeta$$ (3)

Equations 1, 2 are measurement models, and Equation 3 are structural models. X is the observation variable of exogenous potential variables, that is, a series of indicators to measure the objective built environment, subjective perception and individual attributes; ξ is the potential exogenous variable. Y is the observed variable of endogenous potential variable; *η* is the endogenous latent variable. Λ_x_ and Λ_y_ are the factor load matrices of the variables X and Y. β and *Γ* are both path coefficients, β is the relationship between endogenous latent variables. Γ is the effect of exogenous latent variable on the value of endogenous latent variable. ζ is the error term of the structural equation (Table 1).

**Table 1:** Definition of variables.

Latent variable	*n*.	Observed variable	Variable interpretation
Individual attribute feature	ER	ER1 Age	Gap filling
		ER2 Educational level	Primary and below = 1 Junior high school = 2 High school/secondary vocational school = 3 Junior college = 4 Undergraduate course = 5 Master = 6 Learned scholar = 7
		ER3 Duration of residence	0–4 years = 1 5–9 years = 2 10 years or more = 3
		ER4 Monthly income (RMB)	0 yuan = 1 1–2,200 yuan = 2 2,201–5,000 yuan = 35,001–10,000 yuan = 4 10,001–30,000 yuan = 5 More than 30,000 yuan = 6
		ER5 Think of your family’s financial situation	Very poor = 1 Relatively poor = 2 Middle income = 3 Relatively rich = 4 Very rich = 5
Transportation	TF	TF1 Walking comfort	Strongly disagree = 1 Disagree = 2 General = 3 Approval = 4 Strongly agree = 5
		TF2 Lane design is reasonable	
		TF3 Life circle traffic environment	
Living business	LF	LF1 Sports exercise facilities	Strongly disagree = 1 Disagree = 2 General = 3 Approval = 4 Strongly agree = 5
		LF2 Life circle business environment	
		LF3 Life circle catering environment	
		LF4 Life circle medical environment	
		LF5 Life circle education environment	
Cultural and leisure	CF	CF1 Children’s playground in the community	Strongly disagree = 1 Disagree = 2 General = 3 Approval = 4 Strongly agree = 5
		CF2 Leisure activities in the community	
		CF3 Cultural activities in community	
		CF4 Living circle leisure environment	
		CF5 Cultural environment of life circle	
Female-friendly	WF	WF1 Child-friendly in community	Strongly disagree = 1 Disagree = 2 General = 3 Approval = 4 Strongly agree = 5
		WF2 Female-friendly in community	
		WF3 Life circle child-friendly environment	
		WF4 Night lighting	
Perception	SP	SP1 Property Service Level	Strongly disagree = 1 Disagree = 2 General = 3 Approval = 4 Strongly agree = 5
		SP2 Neighborhood	
		SP3 Cell Governance	
Satisfaction	S	S1 Satisfaction in community	Strongly disagree = 1 Disagree = 2 General = 3 Approval = 4 Strongly agree = 5
		S2 Satisfaction in life circle	
		S3 Satisfaction with current living conditions	

### 3.3 Data analysis

Table 2 describes each dependent variable, latent variable, observed variable and variable index. From the perspective of women, based on the existing evaluation methods of community living satisfaction (34–38), a five-level scale is adopted to comprehensively consider subjective and objective conditions, and then a structural model is formed.

**Table 2:** Basic information of female residents.

Dimension	Frequency	%
Age		
19 ~ 29	264	28.6
30 ~ 39	433	46.9
40 ~ 50	226	24.5
Education		
Primary and below	3	0.3
Junior high school	29	3.1
High school/Secondary vocational School	56	6.1
Junior college	142	15.4
Undergraduate	507	54.9
Master	167	18.1
Doctor	19	2.1
Residential time		
0 ~ 4 years	369	39.9
5 ~ 9 years	258	28.0
10 years and above	296	32.1
Monthly income (RMB)		
0	26	2.8
1 ~ 2,200	30	3.3
2,201 ~ 5,000	109	11.8
5,001 ~ 10,000	392	42.5
10,001 ~ 30,000	323	35.0
30,000 above	43	4.7
Perception of economic situation		
Very poor	8	0.9
Relatively poor	96	10.4
Middle income	709	76.8
Relatively rich	98	10.6
Strong rich	12	1.3

As far as the built environment of facilities is concerned, the classification standard of community facilities is adopted, which is divided into three categories: transportation facilities, living and commercial facilities, and cultural and leisure facilities. Among them, the walking comfort level (TF1) in the community, the reasonableness of lane design (TF2) and the traffic condition in the living circle (TF3) are the key indicators to evaluate the community transportation facilities (36). The physical exercise facilities (LF1) set in the community and the commercial (LF2), catering (LF3), medical (LF4) and educational (LF5) environments within the living circle play an important role in the evaluation of living and commercial facilities, which profoundly affects the overall perception of female residents on the living environment and the evaluation of life quality (35). At the same time, the construction completeness of children’s amusement facilities (CF1), leisure activity venues (CF2) and recreational activities facilities (CF3) as well as the leisure and cultural environment (CF4,5) in the living circle of the community profoundly reflects the degree of concern about the quality of life of residents and the level of comprehensive livability of a community (37). Child friendliness (WF1,3) can confirm the difference in women’s perception of the sense of security in the community living environment. Night lighting (WF4) reflects a broader social context, namely care and inclusiveness. Bright streets not only reduce the risk of crime but also enable women to participate in night-time social activities and cultivate a sense of community belonging (1). These factors have a profound and unique impact on women’s life experience in the community, especially when they bear the main responsibility of child rearing and face daily travel and social activities (38). The service level of residential properties (SP1), the friendliness of the surrounding neighborhoods (SP2), and the governance situation in the residential community (SP3) greatly affect women’s subjective perception of the living environment, and shape their daily life experience and emotional atmosphere (34).

Before starting the statistical analysis, filter outliers in the data by checking for *Z*-values and missing values to ensure that the results of the statistical test are more accurate. Follow three steps to test the hypothesis. First, a two-step confirmatory factor analysis method was used to evaluate each structure separately and then validate the overall measurement model (39). Secondly, the reliability, convergence validity and discriminant validity of the model are tested to ensure the psychometric properties of the model. Thirdly, the proposed path model is estimated and hypothesis tested.

## 4 Results

### 4.1 Sample and reply brief

As shown in Table 2, the majority of respondents (41.7%) were aged between 30 and 39, followed by residents aged between 19 and 29 (28.6%). They are well educated, with just under 75.1% of them having a university degree or above, with the largest number of residents having a bachelor’s degree (54.9%). The majority of respondents have lived in their communities for 0–4 years (39.9%), which may be strongly related to the migrant worker base in Shanghai, followed by more than 10 years (32.1%). The salary income of the respondents is mostly between 5,000 and 9,999 yuan (42.5%), followed by 10,000 to 19,999 yuan (35.0%), which may be related to Shanghai’s geographical advantages and economic development. In addition, residents’ awareness of the level of family economic status shows a strong normal distribution law, and the majority of middle income is 76.8%. These statistics show distinctive characteristics on all sides. Age, education, length of residence and the distribution of wages and income are all consistent. These detailed data accurately reflect the current situation of Shanghai. Economically, the proportion of high income corresponds to its developed degree; In terms of education, the proportion of high education shows its educational advantages, which has been scientifically verified and has high statistical significance.

### 4.2 Structural equation model

**Figure 3:**
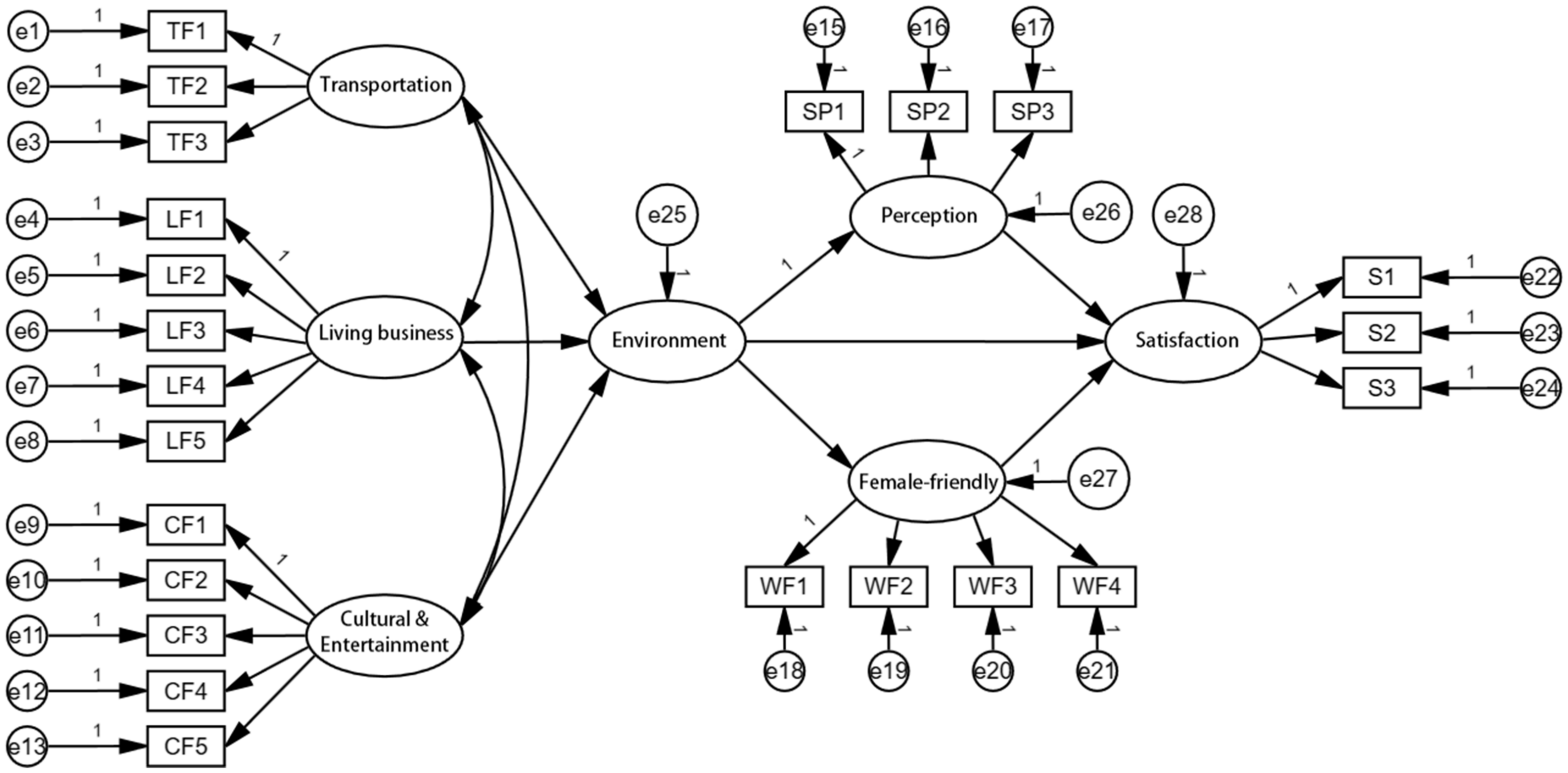
Measurement model data.

**Figure 4:**
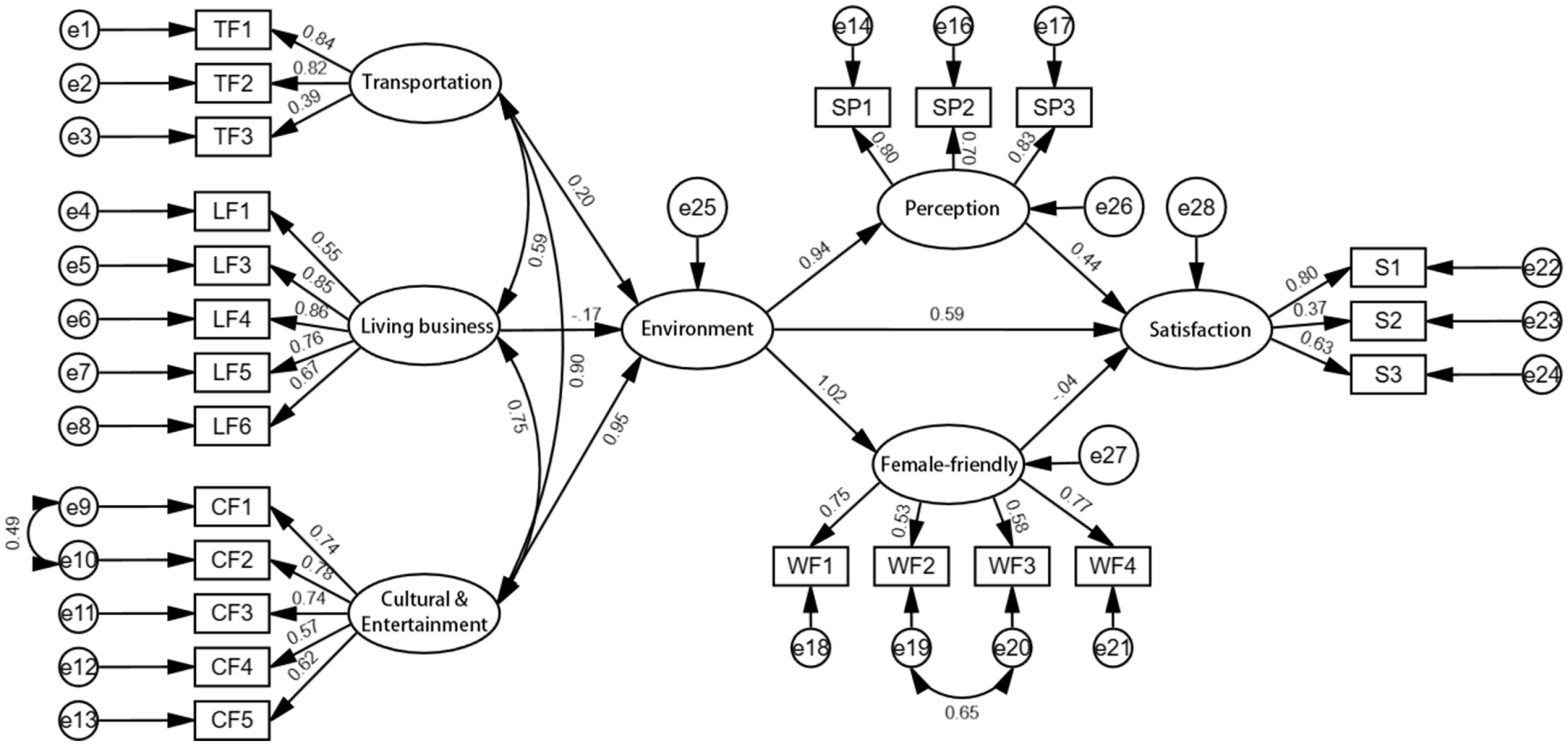
The coefficients of each path in the measurement model.

Before investigating the hypothesized relationships among the latent variables, the proposed factor structure is validated using confirmatory factor analysis (CFA). According to the suggestion of Anderson and Gerbing (40), a two-step method was adopted to verify the multi-attribute scale. The first step is to verify the measurement model for each structure, followed by testing the entire measurement model. The combination reliability and validity of structural equation model are acceptable through confirmatory factor analysis. Each fitting index of the final model is within the acceptable range (Table 3), so the structural equation model hypothesis is accepted. The results of structural equation models designed in this paper are satisfactory, and the chi-square to freedom ratio (*x*^2^/df) is less than 3. The approximate root-mean-square error RMSEA is less than 0.08, indicating that the model has a good data fit (41). Although the values of GFI, AGFI, CFI and TLI did not fully reach the ideal value, they were all greater than 0.8, which was within the acceptable range. It indicates that the model and data fit well (42) (Figures 3, 4).

**Table 3:** Model fit index.

Model	*x*^2^/df	RMSEA	CFI	AGFI	GFI	TLI	IFI
Index	<3	<0.080	>0.9	>0.9	>0.9	>0.9	>0.9
Actual	1.358	0.034	0.992	0.971	0.984	0.991	0.990

As shown in Table 4, at the reliability level, a scale reliability analysis was conducted on the experimental data. The Cronbach’s *α* coefficient of the overall scale was 0.949 (>0.80 threshold), and the single-item α coefficients of all 25 observed variables exceeded 0.80, demonstrating the outstanding internal consistency of the measurement tool (43). At the validity level, dimensionality reduction factor analysis was conducted on the experimental data. The suitability value of KMO sampling was 0.952 (>0.80, *p* < 0.001), strongly supporting the suitability of the data for factor analysis (44).

**Table 4:** Reliability and validity test results of latent variables.

Latent variable	C. R.	AVE.	Cronbach alpha
Transportation (TF)	0.630	0.831	0.803
Living business (LF)	0.552	0.877	0.813
Leisure and Entert. (CF)	0.604	0.883	0.836
Female-Friendly (WF)	0.649	0.880	0.812
Perception (SP)	0.738	0.894	0.819
Satisfaction (S)	0.523	0.810	0.780

### 4.3 Analysis of influencing factors of facility/life satisfaction

As shown in Table 5, the research found that the overall satisfaction of female residents with the community (S1) was 3.76, which is greater than the median value of 3. This result indicates that female residents generally consider their living conditions to be “fairly satisfactory.” From the perspective of sample distribution, “Strongly agree,” “Approval,” “General,” “Disagree,” and “Strongly disagree” accounted for 24.1, 39.1, 29.0, 4.7%, and only 3.1%, respectively. The standard deviation of the overall satisfaction within the community was 0.972, indicating that the choices of most female residents were similar. Meanwhile, the overall satisfaction of female residents with the living circle (S2) was 3.83, slightly higher than that within the community, suggesting that female residents are more satisfied with the environment within the living circle than that within the community. Therefore, in terms of the overall satisfaction of female residents, they are generally satisfied with the overall environmental quality of the community.

**Table 5:** Satisfaction evaluation of facilities.

Variable	Observation variable	Mean	Standard deviation	Principal index mean
TF	TF1	3.58	1.032	3.61
	TF2	3.22	1.142	
	TF3	4.02	0.800	
LF	LF1	3.55	1.050	3.83
	LF2	3.95	0.781	
	LF3	3.90	0.804	
	LF4	3.90	0.777	
	LF5	3.84	0.779	
CF	CF1	3.37	1.126	3.50
	CF2	3.55	1.088	
	CF3	3.33	1.074	
	CF4	3.86	0.832	
	CF5	3.37	0.946	
S	S1	3.76	0.972	3.76
	S2	3.83	0.754	
	S3	3.68	0.843	
WF	WF1	3.27	1.019	3.34
	WF2	3.19	0.998	
	WF3	3.41	0.947	
	WF4	3.47	1.073	

Regarding the degree of female-friendliness (WF2), female residents’ perception of this aspect within the community was relatively poor at 3.19, with a standard deviation of 0.998, indicating that the views of the respondents were relatively concentrated but not completely uniform. Thus, in terms of the current infrastructure in Yangpu District, the degree of female-friendliness is not high. Urban builders and decision-makers can take this as a starting point to enhance the overall satisfaction of female residents with their living environment. At the same time, the concept of child-friendly communities regards child-rearing as a shared social responsibility, breaks the traditional constraints on women, changes the social perception of the roles of men and women in child-rearing, enhances women’s social status, and promotes the improvement of the degree of female-friendliness. The research found that the degree of child-friendliness within the living circle (3.41) was higher than that within the community (3.27), indicating that there is still potential for child-friendliness within the community to be explored. Finally, night lighting (WF4) can reflect female residents’ experience of community safety from the side. Adequate lighting can increase the usage rate and comfort of parks, squares, etc., promote the balanced utilization of public resources, and create a female-friendly atmosphere. In contrast, poor lighting may lead to situations where women avoid using these spaces.

Research findings indicate that from a female perspective, the degree of completion and improvement of community supporting facilities has a significant impact on subjective perception (Table 6). Safety-related supporting facilities ensure women’s travel and safety, comfortable environments enhance a sense of ease, and various activity areas catering to different age groups promote family relationships and intergenerational interaction. All these factors lead women to give higher evaluations of the completeness of community supporting facilities, strengthening their sense of identification and subjective perception toward the community. The correlation between the degree of female-friendliness and residential satisfaction is not significant (−0.040). At the same time, subjective perception shows a certain correlation with the built environment of facilities. A good built environment of facilities makes residents feel visually comfortable and pleasant, and use facilities conveniently and efficiently, thereby generating positive subjective perception. In addition, when the built environment of facilities meets the actual living needs of residents and meets or exceeds their functional expectations, residents will give higher evaluations (45). Finally, a safe and comfortable environment will also enhance residents’ living comfort and their identification and affection for the living environment, which is reflected in higher subjective perception evaluations.

**Table 6:** Coefficient estimates for SME.

Path information	Estimate	S. E.	C. R.	*P*
Environment ← Transportation	0.204	0.096	1.884	*
Environment ← Leisure and entertainment	0.950	0.136	6.510	***
Environment ← Life business	−0.173	0.072	−3.121	**
Perception ← Environment	0.941	0.043	24.314	***
Female-friendly ← Environment	1.000	–	–	–
Satisfaction ← Perception	0.444	0.124	3.206	***
Satisfaction ← Environment	0.591	0.338	1.731	**
Satisfaction ← Female-friendly	−0.040	0.361	−0.124	0.902
TF1 ← Transportation	1.000	–	–	–
TF2 ← Transportation	0.843	0.037	29.477	***
TF3 ← Transportation	0.820	0.030	11.867	***
LF1 ← Life business	1.000	-	-	-
LF2 ← Life business	0.854	0.065	17.487	***
LF3 ← Life business	0.864	0.068	17.511	***
LF4 ← Life business	0.767	0.062	16.525	***
LF5 ← Life business	0.673	0.059	15.235	***
CF1 ← Leisure and entertainment	1.000	–	–	–
CF2 ← Leisure and entertainment	0.782	0.030	34.224	***
CF3 ← Leisure and entertainment	0.746	0.041	23.268	***
CF4 ← Leisure and entertainment	0.578	0.032	17.528	***
CF5 ← Leisure and entertainment	0.629	0.037	19.139	***
SP1 ← Perception	1.000	–	–	–
SP2 ← Perception	0.885	0.033	22.865	***
SP3 ← Perception	0.837	0.034	28.294	***
WF1 ← Female-friendly	1.000	–	–	–
WF2 ← Female-friendly	0.538	0.042	16.404	***
WF3 ← Female-friendly	0.587	0.040	18.121	***
WF4 ← Female-friendly	0.774	0.044	24.679	***
S1 ← Satisfaction	1.000	–	–	–
S2 ← Satisfaction	0.378	0.033	10.921	***
S3 ← Satisfaction	0.633	0.034	20.025	***

*p < 0.05, **p < 0.01, ***p < 0.001, the path “←” represents the built environment latent variable represented by the corresponding explicit variable.

In the demonstration of the indirect effect, it was found that the direct influence quantity of the “Environment → Perception → Satisfaction” path was 0.444, the indirect influence quantity was 0.941*0.444 = 0.418, and the total influence was 0.862 (*p* < 0.001). The direct impact of the “Environment → Satisfaction” path was 0.591 (*p* < 0.002). Studies have shown that the built environment has a significant indirect impact on residential satisfaction through subjective perception (*β* = 0.418, 95% CI[0.321, 0.532]). This indicates that 41.8% of the total impact of the built environment on satisfaction is mediated by the subjective perception of residents.

Interestingly, the research found that recreational and entertainment facilities are more attractive to women than transportation and commercial living facilities. Recreational and entertainment facilities can provide women with richer emotional experiences and opportunities for self-actualization. Gyms, dance studios, and other places not only allow women to exercise and maintain their figure but also serve as platforms for them to pursue self-improvement and challenge their limits. In this process, women can feel the enhancement of their own strength and the expansion of their abilities, and gain a sense of achievement and confidence. Museums, theaters, and other cultural and entertainment venues can meet women’s spiritual needs, allowing them to immerse themselves in different emotional worlds and pursue various emotions such as romance, fantasy, and emotion. In contrast, transportation facilities mainly meet basic travel needs and have a single function, making it difficult to bring rich emotional experiences and social value to women; commercial living facilities, although they can meet shopping and consumption needs, are not as significant as recreational and entertainment facilities in terms of emotional satisfaction and social interaction.

With residence time (ER3) as the factor and satisfaction (S1-4) as the dependent variable, the team adopted the single factor ANOVA test method to effectively analyze the influence of a single factor on the dependent variable. The correlation between family life satisfaction and residence time was statistically significant, and the *p*-value was 0.005 (Table 7). As shown in Table 8, pairwise comparison of the data of different groups is carried out to accurately identify the groups with significant differences. The results showed that female residents who lived for more than 10 years had the highest degree of satisfaction with family life compared with those who lived for 0–4 years and 5–9 years. In order to further refine the research results and clarify the differences in family life satisfaction of female residents in different residence periods, the research team conducted multiple comparative post-mortem tests (LSD). The results reveal a positive relationship between the increase of residence time and female residents’ satisfaction with family life, that is, with the extension of residence time, female residents’ satisfaction with family life shows an increasing trend.

**Table 7:** Comparison between residence time and satisfaction.

Observed variable	ER3	Sum of squares	Mean square	*F*	*P*
S1	Interclass	0.509	0.254	0.269	0.764
	Intraclass	870.529	0.946		
S2	Interclass	1.152	0.576	1.013	0.364
	Intraclass	523.143	0.569		
S3	Interclass	3.204	1.602	2.259	0.105
	Intraclass	652.419	0.709		
S4	Interclass	5.031	2.516	5.396	0.005
	Intraclass	428.930	0.466		

**Table 8:** Single-factor ANOVA postmortem test.

Variable	Length of residence (I)	Length of residence (J)	Mean difference (I-J)	S. E.	*P*
S4	0–4	5–9	0.039	0.055	0.484
		10 above	−0.139*	0.053	0.009
	5–9	0–4	−0.039	0.055	0.484
		10 above	−0.177*	0.058	0.002
	10 above	0–4	0.139*	0.053	0.009
		5–9	0.177*	0.058	0.002

*The significance level of the mean difference was 0.05.

### 4.4 Characteristics of female polymer site

**Figure 5:**
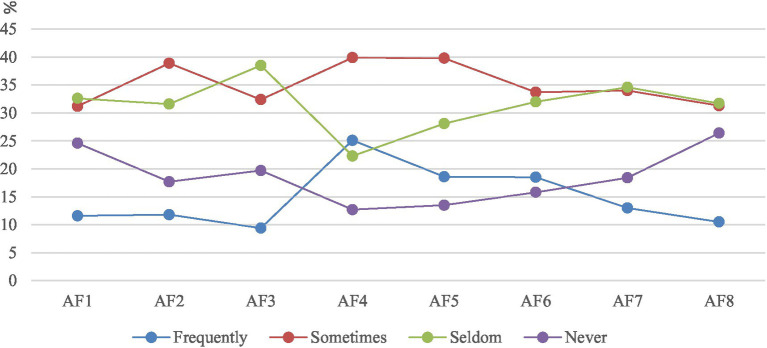
Schematic representation of female gathering points.

As shown in Table 9 and Figure 5, the study found that women always gathered in the shopping malls around the community (25.1%), followed by the nearby parks (18.6%) and cafes/book bars/bars/Internet celebrities punching points (18.5%), while the fitness facilities in the community (9.4%) were the areas where women gathered least. Further analysis shows that the reason why shopping malls have become women’s favorite gathering place may be because they can meet a variety of consumer needs, from daily shopping to fashion and beauty, from catering and entertainment to parent–child activities, providing women with a one-stop leisure experience place. The high concentration of nearby parks reflects women’s love for the natural environment and outdoor activities. Walking in the park and appreciating nature can both relax the body and mind and help maintain health and vitality (46). The appeal of cafes/book bars/bars/online celebrity check-in points may be due to their unique atmosphere and social attributes, which provide women with a warm space to meet friends, exchange emotions and share their lives, while also satisfying their pursuit of fashion, culture and novel experiences.

**Table 9:** Results of female gathering points.

Aggregation type/frequency	Never	Seldom	Sometimes	Frequently
AF1 Children’s activity place in the community	24.6%	32.6%	31.2%	11.6%
AF2 Leisure activities in the community	17.7%	31.6%	38.9%	11.8%
AF3 Fitness facilities in the community	19.7%	38.5%	32.4%	9.4%
AF4 Shopping malls around the community	12.7%	22.3%	39.9%	25.1%
AF5 The neighborhood is surrounded by parks	13.5%	28.1%	39.8%	18.6%
AF6 Neighborhood cafe/book bar/bar/Internet celebrity punch point	15.8%	32.0%	33.7%	18.5%
AF7 Neighborhood beauty/manicure/hairdressing	18.4%	34.6%	34.0%	13.0%
AF8 Neighborhood fitness/dance/yoga	26.4%	31.7%	31.3%	10.5%

In contrast, the low aggregation rate of fitness facilities in the community suggests some potential problems. On the one hand, it may be that the types of fitness facilities are relatively simple and outdated, which cannot meet the diversified fitness needs of women, such as the lack of special equipment suitable for women such as yoga and pilates, or the limited space of the venue and overcrowding during peak hours, which affect the use experience of women (47). On the other hand, the lack of publicity and promotion may also be one of the reasons, many women may not know the specific functions and opening hours of the fitness facilities in the community, resulting in these facilities are not fully used. In order to improve the utilization rate of fitness facilities in the community, community managers can consider increasing the diversity and interest of facilities, regularly maintaining and updating equipment, holding some fitness activities or courses, attracting more women to participate in fitness activities in the community, promoting intergenerational communication and interaction, and creating a more healthy, harmonious and dynamic community atmosphere.

## 5 Discussion

This study takes 36 communities in Yangpu District, Shanghai as the research objects and explores the relationship between the built environment of residential supporting facilities and the friendliness of women, subjective perception, and residential satisfaction from the perspective of women. The research results not only enrich the empirical research on “female-friendly cities” in the Chinese context, but also provide a new perspective on the micro-mechanisms by which community facilities shape women’s life experiences.

### 5.1 Mechanism of facility environment and female experience

The core finding of this study is that the built environment of community facilities significantly affects female-friendliness and residential satisfaction, which is consistent with and expands on existing theories of social infrastructure. Urban social infrastructure (such as parks and commercial spaces) is crucial for promoting social interaction and a sense of belonging (24, 53). The research further clarifies this relationship by focusing on the female group, revealing that facilities are not merely functional Spaces but carriers of gender needs: well-designed recreational facilities, safe lighting, and child-friendly areas directly enhance women’s sense of being cared for, thereby strengthening their sense of identity with the community.

It is worth noting that recreational and entertainment facilities are more attractive to women than transportation or commercial facilities. This finding is consistent with the qualitative study by Peng et al. (10), which emphasizes that women’s activity preferences are closely related to emotional experiences and social interactions. Gyms, community activity centers and parks not only meet physical needs, but also provide platforms for self-development and interpersonal connections. In contrast, transportation facilities mainly meet basic travel needs, while commercial facilities focus on utilitarian consumption and have a relatively small impact on shaping women’s emotional attachment to the community.

Another notable result is that there is no significant direct correlation between female-friendliness and residential satisfaction. Resident satisfaction is a multi-dimensional structure, influenced by basic living conditions and background factors (13). For women, although facilities that are friendly to women (such as mother-and-baby rooms and night lighting) are important, they may be secondary to core needs such as affordable housing or convenient commuting. Furthermore, the heterogeneity of female needs reflects that young single women prioritize safety and married mothers focus on childcare facilities (48), which may dilute the unified impact of female-friendliness on overall satisfaction.

### 5.2 Guiding women-friendly community construction

The research results have a clear impact on urban planners and decision-makers who aim to improve the quality of life for women. The first is to give priority to optimizing leisure and entertainment facilities. Given the strong appeal of the community to women, it should expand diverse recreational Spaces such as yoga studios, book bars and parent–child activity areas, and ensure that these Spaces are convenient, safe and well maintained. This is in line with the initiative to build “women-friendly cities” and can be incorporated into local policies through incentives (9). Second, give full play to the intermediary role of subjective perception. Research has confirmed that the built environment indirectly affects residents’ satisfaction through their perception. Therefore, improving the “soft environment” is as important as upgrading the hardware. For instance, organizing women’s cultural salons or volunteer activities can enhance their sense of belonging, even in communities with limited facilities and resources. Third, build female-friendly facilities in a targeted manner. As women’s needs vary by age, family structure and lifestyle, a one-size-fits-all strategy is ineffective. For instance, communities with more young mothers should give priority to mother-and-baby rooms and children’s playgrounds, while those with older women may need fitness trails and social lounges. Surveys that regularly assess the heterogeneity of demand will guide precise facility upgrades.

### 5.3 Limitations and future research

At the same time, there are some shortcomings in this study. First, due to the limitations of data acquisition approaches and methods, the selection of communities and indicators in Yangpu District of Shanghai is limited, and the current infrastructure situation in the residential area cannot be fully explained. Second, Yangpu District is selected as the research site in this paper, and the research scope can be expanded to include Pudong, Huangpu, Changning and other districts and counties in the future, so that the samples will be more comprehensive and objective. Third, this paper mainly uses cross-sectional data, which cannot well reveal the longitudinal mechanism of the impact of the built environment and subjective perception evolution of facilities within the scope of daily activities on the overall site satisfaction.

## 6 Conclusion

### 6.1 Hypothesis testing

As shown in Table 8, this paper carefully formulated five hypotheses around the issues related to concentrated housing, and based on the solid field investigation and detailed questionnaire data of 36 residential communities in Yangpu District, carried out in-depth analysis, and finally reached a clear and revelatory conclusion. After rigorous analysis, four hypotheses were established with strong data support, and another hypothesis was falsified. Specifically, there is no direct correlation between female friendliness in the community and residential satisfaction, and the path coefficient between the two is −0.04, which cannot directly constitute the correlation hypothesis, so hypothesis 1 is falsified (Table 10).

**Table 10:** Presents the results of the model.

Hypothetical path	Estimate	Testing
H1: Satisfaction ← Female-friendly	−0.04	Not support
H2: Female-friendly ← Environment	1.02	Support
H3: Perception ← Environment	0.94	Support
H4: Satisfaction ← Environment	0.59	Support
H5: Satisfaction ← Perception	0.44	Support

There is a strong correlation between the quality of the built environment of the facility and the friendliness of women. Women can feel the community’s care for women by using the facilities in daily life. When there are sufficient and reasonably arranged lighting facilities around the community, women will feel more secure when traveling at night, reflecting the community’s attention to women’s safety needs (49). The details of public restrooms also reflect the friendliness of women (50). A sufficient number of women’s toilets and clean, well-equipped sanitation facilities can avoid embarrassing queues for women in public places, reflecting respect for and care for women’s physiological needs. The presence of the mother and baby room provides great convenience for nursing mothers, such as comfortable nursing environment and complete maternal and baby supplies, so that mothers can calmly take care of their children when they go out and feel the support and care of the community for nursing women (51), thus enhancing their sense of belonging and identity to the community. Therefore, hypothesis 2 is valid.

The quality of the built environment of the facility greatly affects the subjective perception and experience. High-quality facility environment can bring pleasure, comfort and convenience to women and improve their cognition of the quality of life. On the contrary, poor facility environment will cause negative emotions and reduce women’s subjective perception of living environment, especially in commercial areas, so hypothesis 3 is valid. At the same time, the built environment and subjective perception of facilities have a certain impact on women’s residence satisfaction. Humanized facilities environment, for example, the community is equipped with well-maintained fitness facilities, safe and comfortable children’s play area, bright and clean public space, etc., can improve the convenience and comfort of women’s daily life, so that they feel the quality of life and community care in the actual use of the process, and then enhance the recognition of the living environment. In terms of subjective perception, women’s perception of community atmosphere, harmony of neighborhood relationship and community safety also affect their living satisfaction to a large extent (39, 52). A community full of friendly atmosphere, neighbors helping each other and good security will make women have a strong sense of belonging and security in psychology. Even if the facilities are not perfect, they can make up for some deficiencies by positive subjective feelings, which is different in the new and old residential communities. On the contrary, if there are defects in the construction of facilities, coupled with adverse subjective perception factors, such as apathetic interpersonal relations in the community, serious noise pollution, etc., it will greatly reduce women’s satisfaction with living, and may even lead to their idea of moving out, so hypothesis 4 and hypothesis 5 are valid. Therefore, in the process of improving women’s living satisfaction, it is necessary to pay attention to the optimization of the built environment of facilities and the creation of positive subjective perception, which complement each other and jointly create an ideal living space for women.

### 6.2 Suggestions for management and builders

According to the experimental data and conclusions obtained in this paper, the following suggestions can be provided for the managers and policy makers of Shanghai urban construction. First of all, in terms of planning and policy formulation, in view of the low perception of female residents on the female-friendly degree of residential areas in Yangpu District, policy documents are issued to encourage and guide the construction of female-friendly supporting facilities in residential areas, such as giving tax incentives and plot ratio incentives to developers or properties that add female-only facilities. Set up a special fund to subsidize the renovation of women-friendly facilities in old residential areas to increase the enthusiasm of builders and managers. At the same time, the evaluation index system of supporting facilities in female-friendly residential areas should be established, and the facilities construction in each residential area should be quantitatively evaluated on a regular basis, and the evaluation results should be published to the public.

Secondly, in terms of optimizing the rationality of facility layout, the layout of supporting facilities in residential areas should be rationally planned, and the principles of convenience, accessibility and safety should be followed. Business service facilities, medical and health facilities, educational facilities, etc. are centrally arranged in the center of the residential area or near the main entrances and exits to form a convenient life service circle and reduce the travel distance and time cost of female residents; Placing women-only facilities in a visible, easy-to-find, relatively separate and private location while ensuring a safe and comfortable surrounding environment; Pay attention to the connectivity between different supporting facilities, through the walking path, bicycle path and other green transportation means to organically connect the facilities, so as to facilitate the conversion and passage of female residents between different facilities.

Finally, in terms of the establishment of facility operation and management mechanism, based on the characteristics reflected in the female cluster site, a sound operation and management system and service norms for supporting facilities in the residential area can be formulated, and the operation subjects, management responsibilities, service content and charging standards of the facilities can be clarified. For commercial service facilities, we should strengthen investment management, introduce businesses that meet women’s consumption needs and quality requirements, and provide diverse and high-quality goods and services for female residents. For public service facilities, such as community activity centers, libraries, gyms, etc., the opening hours should be reasonably arranged, the utilization rate of the facilities should be improved, and professional management and service personnel should be equipped to ensure the normal operation and service quality of the facilities.

## Data Availability

The original contributions presented in the study are included in the article/supplementary material, further inquiries can be directed to the corresponding author.
